# Upgrading Supply Chain Management Systems to Improve Availability of Medicines in Tanzania: Evaluation of Performance and Cost Effects

**DOI:** 10.9745/GHSP-D-16-00395

**Published:** 2017-09-27

**Authors:** Marasi Mwencha, James E Rosen, Cary Spisak, Noel Watson, Noela Kisoka, Happiness Mberesero

**Affiliations:** a John Snow, Inc., Dar es Salaam, Tanzania.; b Avenir Health, Washington, DC, USA.; c John Snow, Inc., Arlington, VA, USA.; d Ops Mend, Boston, MA, USA.

## Abstract

**Background:**

To address challenges in public health supply chain performance, Tanzania invested in a national logistics management unit (LMU) and a national electronic logistics management information system (eLMIS). This evaluation examined the impact of those 2 key management upgrades approximately 1 year after they were introduced.

**Methods:**

We used a nonexperimental pre-post study design to compare the previous system with the upgraded management system. We collected baseline data from August to November 2013. We conducted round 1 of post-implementation data collection during April and May 2015, about 1 year after implementation of the upgrades. We evaluated key indicators of data use and reporting; supply chain management practices such as storage and supervision; supply chain performance including stock-out and expiry rates; and supply chain cost and savings. We analyzed the data using a range of techniques including statistical testing of baseline and round-1 results, and cost, cost-effectiveness, and return on investment analysis.

**Results:**

The upgrades were associated with improvements in data use, accessibility, visibility, and transparency; planning, control, and monitoring; support for quantification; stock-out rates; stock-out duration; commodity expiry; and forecast error. The upgraded system was more costly, but it was also more efficient, particularly when adjusting for the performance improvements. The upgrades also generated substantial savings that defrayed some, but not all, of the investment costs.

**Conclusion:**

Upgrades to Tanzania's supply chain management systems created multiple and complex pathways to impact. One year after implementation, the LMU and eLMIS brought about performance improvements through better data use and through improvements in some, but not all, management practices. Furthermore, the upgrades—while not inexpensive—contributed to greater system efficiency and modest savings.

## Abstract (precis)

Investments in a national logistics management unit and electronic logistics management information system resulted in better data use and improvements in some, but not all, management practices. After 1 year, key improvements included reduced stock-out rates, stock-out duration, and expiry rates. Although the upgraded systems were not inexpensive, they contributed to greater system efficiency and generated modest savings that defrayed much of the investment and maintenance costs.

## INTRODUCTION

Universal health coverage includes access to safe, effective, high-quality, and affordable essential medicines and vaccines for all.^1^ In Tanzania, the steadily improving performance of the public health supply chain has contributed to a dramatic reduction in mortality from HIV, malaria, and tuberculosis^2^ for vaccine-preventable deaths,^3^^,^^4^ and expanded access to contraception.^5^ Nonetheless, in recent decades, the complexity of the public health supply chain, and the volumes and varieties of health care products, have greatly increased. This has resulted in a fragmented system that has been difficult to coordinate and has hampered product availability.^6^

In 2014, to address these challenges, Tanzania made a major investment to upgrade its management systems for the public health supply chain. The government established a national logistics management unit (LMU) to oversee all key public health commodities by organizing, monitoring, and supporting all supply chain activities within all the logistics systems in the country. The LMU centralizes and harmonizes the management of disparate program supply chains, promotes greater efficiency in supply chain management, and ensures better customer service.^7^ Tanzania also introduced a national web-based electronic logistics management information system (eLMIS) to support the aggregating, reporting, and visualizing of data collected from the paper-based system. Several other countries have converted from a paper-based to an electronic system.^8^ This article describes the results from an evaluation of these key management upgrades approximately 1 year after introduction.

Tanzania invested in 2 supply chain system upgrades—a national logistics management unit and an electronic logistics management information system.

In Tanzania, the Ministry of Health, Community Development, Gender, Elderly and Children (MOHCDGEC) has overall responsibility for the public health supply chain that serves more than two-thirds of Tanzania's 49 million people.^9^ This supply chain includes the quasi-autonomous Medical Stores Department (MSD), which has its headquarters in the capital of Dar es Salaam and operates 9 zonal stores. It also includes MOHCDGEC-operated facilities, consisting of 20 regional vaccine stores, 137 district stores, and 5,500 service delivery points (SDPs)—hospitals, health centers, and health posts—where clients get their medicines and vaccines. The value of medicines and other commodities moving through the public health supply chain each year is around 300 billion Tanzanian shillings (TSh) (about US$200 million at 2013 exchange rates).

**FIGURE 1:**
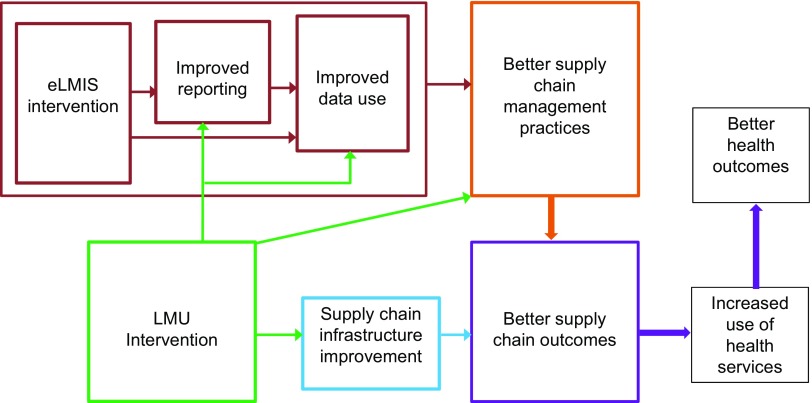
Conceptual Framework for the Management Upgrade Interventions Abbreviations: eLMIS, electronic logistics management information system; LMU, Logistics Management Unit.

We hypothesized that the MOHCDGEC implementation of the upgraded management systems would affect performance and, ultimately, health outcomes, in several ways (Figure 1). The LMU would contribute to improvements in reporting and data management; management practices through consolidation of oversight; supply chain infrastructure; and supply chain outcomes. The eLMIS would enhance management through improved reporting and data use.

## METHODS

### Study Design

We used a nonexperimental pre-post study design to compare the previous system with the upgraded management system. We could not use an experimental design because the eLMIS was not piloted before the introduction. Baseline data collection took place from August to November 2013. A post-implementation round 1 of data collection took place in April and May 2015, approximately 1 year after implementation.

We analyzed supply chains for 2 commodity groups that account for most of the throughput in Tanzania: (1) antiretroviral drugs and HIV tests, and (2) reproductive health, malaria, and essential medicines that form the integrated logistics system (ILS). We also examined a subset of 38 commodities for many of the management and supply chain performance indicators (Supplement 1).

### Data Collection and Measures

#### Survey Instruments

We used 3 types of survey instruments to collect data: distribution and dispensing of commodities survey, performance and cost survey, and data use and management practice survey (Table 1).

**TABLE 1.** Survey Instruments

Survey	Sample	Timing
Distribution and dispensing of commodities	Nationally representative sample of 220 SDPs	Baseline: August, October 2013 Round 1: April 2015
Performance and cost	17 district offices 9 MSD zonal stores MSD headquarters	Baseline: October 2013 Round 1: April 2015
Data use and management practice	16 central-level supply chain stakeholders	Baseline: October–November 2013 Round 1: May 2015

Abbreviations: MSD, Medical Stores Department; SDPs, service delivery points.

We used surveys to collect data on supply and distribution, performance and cost, and data use and management practices.

The distribution and dispensing of commodities survey collected data from a nationally representative sample of 220 health facilities at baseline in August and October 2013, and in April 2015 for the round 1 analysis. To develop this survey, we repurposed End-Use Verification (EUV) surveys, which monitor stock availability and dispensation of ILS commodities on a quarterly basis across a nationally representative sample of health facilities. We added questions on additional performance indicators and cost and expanded the focus to HIV commodities.

We administered the performance and cost survey in October 2013 and April 2015 at 17 district offices, 9 MSD zonal stores, and MSD headquarters. This survey collected information related to data reporting, data use, supply chain management practices, and supply chain outcomes.

The third survey was on data use and management practices, which we administered to central-level supply chain stakeholders, including the MOHCDGEC, development partners, and implementing technical assistance partners. At baseline in October and November 2013, we conducted 14 such surveys at the central level, including 3 for the HIV program, 5 for malaria, 3 for essential medicines, and 3 for family planning and maternal and child health (MCH). During round 1 in May 2015, we conducted 16 surveys at the central level, including 4 for the HIV program, 5 for malaria, 3 for essential medicines, and 4 for family planning and MCH.

We also gathered national-level data from in-person interviews with individual supply chain stakeholders, including government staff, development partners, and technical assistance agencies staff who are considered to be some of the primary users of data, and a review of performance and financial databases.

#### Additional Data Sources for Supply Chain Outcomes

In addition to the surveys, we collected data from MSD's enterprise resource planning system (Epicor 9) and reports on forecast and actual consumption to measure the impact of the upgrades on key supply chain outcomes including stock-outs, inventory levels, commodity expiries, and consumption forecasts.

#### Total Supply Chain Cost Model

To evaluate the impact on costs, we built a total supply chain cost model for multiple tiers and key supply chain functions. In addition to cost surveys at the district and SDP levels, we relied on financial records for MSD central and zonal costs. We obtained the salary, equipment, vehicle, and other price data from MOHCDGEC and other financial records. We determined the commodity throughput value, defined as the average of receipts and issues, using Epicor 9; to estimate any missing price data, we used various program quantification reports or publicly available international price databases. We valued inputs in the local currency, TSh, or in US dollars, as appropriate.

#### Investment Costs and Savings

We conducted a cost-benefit analysis to measure savings associated with process improvements. Investment cost data came primarily from implementing partners' financial reports. To measure savings, we used data related to lower purchase prices for products and lower expiry.

### Data Analysis

#### Survey Measurements

We analyzed the impact of the LMU and eLMIS management upgrades on reporting by combining indicators of timeliness, quality, and reporting rates into a composite index by product group, scaled from 0 to 100. We averaged the scores across facilities in the same tier and combined them into a single reporting score. Data on the reporting indicators came from the logistics data management and inventory control surveys, an EUV facility survey, and existing databases. For round 1, the eLMIS provided additional data on reporting.

To measure the impact on data use, we constructed a composite index for each dimension—transparency, timeliness, visibility, and accessibility—on a scale from 0 to 100. We calculated these scores based on interviews with the supply chain stakeholders.

We also measured 7 areas of management practice: quantification, storage, transportation, inventory management, logistics data management, monitoring and control, and design and planning. Data for the management practice indicators came from specially designed surveys applied at baseline and round 1 and modeled on the Logistics System Assessment Tool.^10^ We constructed management practice composite indices on a 0 to 100 scale; calculated scores for each facility; then averaged scores across facilities by level, program, and the entire supply chain. We combined the 7 management practice indicators into a single “super-index” for management practice, using weights from a study on health care supply chain personnel.

We evaluated the impact of the upgrades on key supply chain outcomes by measuring the percentage of SDPs stocked out of commodities at the time of the survey; percentage of SDPs stocked out for more than 7 days; and percentage of SDPs with inventory levels below minimum, between minimum and maximum levels, or over maximum.

#### Supply Chain Outcomes

Combining data from the EUV surveys and MSD's enterprise resource planning system, Epicor 9, we measured the average level of commodity expiries as a percentage of annual throughput (Supplement 2). Using reports on forecast and actual consumption, we measured the accuracy of consumption forecasts, defined as the deviation of the forecast from actual consumption, as a percentage of actual consumption.

#### Total Supply Chain Cost Model

Following the standard USAID | DELIVER PROJECT approach,^11^ we built a total supply chain cost model by measuring costs at each tier—central, zonal, district, and SDP—and for each of the 4 key supply chain functions—procurement, storage, transport, and management. We reported results in both TSh and US dollars, at an exchange rate of TSh 1,570 per US dollar. We reported all costs in constant 2013 prices. We valued inputs at their market or economic cost. We extrapolated from survey information to estimate a total national supply chain cost. Combining costs with throughput, we also calculated a cost per unit of throughput value. We conducted a cost-effectiveness analysis that focused on average cost-effectiveness adjusted for supply chain performance. The study compared costs and cost-effectiveness for 2013 with costs and cost-effectiveness for the 1-year period from April 2014 to March 2015.

#### Sensitivity Analysis

Because of limitations in the survey approach, or missing or incomplete data, there was substantial uncertainty around throughput, cost, and performance values. We used a Monte Carlo approach in a sensitivity analysis to help determine the extent to which changes in these values might substantially alter the findings (Supplement 3).

#### Cost-Benefit Analysis

To conduct the cost-benefit analysis, we compared investment costs with savings associated with process improvements. Investment costs were the total value of resources applied toward development and implementation of both the LMU and eLMIS, including one-time upfront costs and ongoing operations costs. We measured savings associated with lower purchase prices for products, lower expiry, and absorption of existing staff, supervision, and training costs. Although designers of the upgrades believed that the eLMIS and LMU would generate savings related to price reduction from fewer emergency orders and lower inventory holding costs, we did not have data to measure these savings.

We used the data related to inventory value, product price, and product expiry quantities to measure and document baseline values and to calculate ratios for each area of expected savings. We measured return on investment by comparing cumulative savings with investment (or cost) over a defined time horizon. To estimate expected net benefit and return on investment, we applied savings rates at 1 year after the upgrades were implemented to the baseline values to calculate expected cost savings from process improvements. For savings beyond the first year after the interventions were implemented, we used estimated values to simulate cost savings.

#### Changes to the Distribution System

It is worth noting that some changes to the supply chain distribution structure occurred separately but simultaneously with the rollout of the LMU and eLMIS. It was not possible to completely disentangle the effects of these broader structural changes on supply chain performance in our analysis. Specifically, the HIV supply chain employed supply chain management assistants before implementing the management upgrades. These assistants provided SDP support for HIV logistics data reporting—support that the ILS did not have. The management upgrades expanded the assistants' management approach across the various program supply chains. In addition, during the baseline year, the supply chain completed a shift from a 4-tier (MSD central, MSD zone, district, SDP) to a 3-tier (MSD central, MSD zone, SDP) distribution approach, with direct delivery between the last 2 tiers. We address this issue further in the discussion on limitations.

### Ethics

All data collection and analysis were conducted according to international principles of maintaining privacy and confidentiality of personal information.

## RESULTS

After investing in the LMU and eLMIS, results showed some improvements to Tanzania's overall supply chain performance after approximately 1 year of implementation; however, the full impact of these interventions remains to be seen. Our findings show that data use improved overall, and some management practices improved. Key supply chain outcomes improved, especially reduction of stock-out rates, stock-out duration, and expiry rates at health facilities. The upgrades also contributed to modest savings and greater system efficiency.

The evaluation showed overall improvements in data use, management practices, key outcomes, cost savings, and greater system efficiency.

### Reporting

Statistical comparison showed no difference in scores for reporting on HIV commodities between the baseline and round 1. For example, at the SDP level, reporting on HIV commodities at baseline was 76 and at round 1, 80; at the district level, the scores were 83 and 70, respectively; and at the zonal store level, 75 at both time points.

For ILS commodities at the SDP and district levels, results showed significantly worse reporting scores—dropping from 79 to 67 at the SDP level and from 90 to 67 at the district level. Conversations with Tanzania field operatives suggested that the reduction in reporting performance was temporary, resulting from the interruption in routines, responsibilities, and relationships accompanying the management upgrades at the SDP and district level. Expectations were that the reporting would return to pre-upgrade levels and would eventually improve. For zonal stores, the reporting scores (71) were similar between baseline and round 1, as with HIV commodities.

### Data Use

Overall scores on the 4 dimensions of data use—transparency, timeliness, visibility, and accessibility—increased for all 4 major commodity groups from baseline to round 1: HIV (from 64 to 79); ILS malaria (from 73 to 83); ILS essential medicines (from 60 to 77); and ILS family planning and MCH(from 49 to 83). Large increases in the data visibility score (that is, whether the data are appropriate for the decision-making needs of the stakeholder) drove the overall increase in data use scores (Table 2).

**TABLE 2.** Data Use Scores, Baseline Versus Round 1, by Product Group

Product Group and Time Period	Data Use Dimension				Average
	Accessibility	Visibility	Timeliness	Transparency	
**HIV**					
Baseline	71	21	62	100	**64**
Round 1	81	81	63	92	**79**
**ILS Malaria**					
Baseline	76	74	72	71	**73**
Round 1	82	88	71	92	**83**
**ILS Essential Medicines**					
Baseline	65	34	71	71	**60**
Round 1	61	100	56	92	**77**
**ILS Family Planning and MCH**					
Baseline	53	51	56	36	**49**
Round 1	86	86	70	92	**83**
**Average**					
Baseline	66	45	65	70	**62**
Round 1	77	89	65	92	**81**

Abbreviations: ILS, integrated logistics system; MCH, maternal and child health.

### Management Practices

#### SDP Level

Scores across SDP management practice areas were similar at baseline and round 1, except the score for storage for HIV commodities, which decreased in round 1 (*P*=.047) (Table 3). At baseline, the general management score incorporated the degree to which facility workers received training and the amount of time spent during supervisory visits. HIV commodities showed a particularly high score of 92 in this area, arguably due to the activities of the supply chain management assistants. The management scores for ILS commodities did not improve, although the assistants' activities were expected to expand to include ILS commodities.

**TABLE 3.** Scores on Management Practice Indicators, Baseline Versus Round 1, by Level and Product Group

Level and Product Group	Management Practice Area										Super Index Score	
	Storage		Inventory Management		Transportation		Logistics Data Management		Other Management^a^			
	Baseline	Round 1	Baseline	Round 1	Baseline	Round 1	Baseline	Round 1	Baseline	Round 1	Baseline	Round 1
**SDP level**												
HIV	80	75**	62	63	—	—	71	72	92	93	77	78
ILS	67	66	68	68	—	—	65	63	67	67	62	67
**District level**^b^												
HIV	75	—	77	—	54	—	46	45	51	75**	63	69
ILS	73	—	77	—	53	—	63	52	67	74	63	66
**Zonal level**												
HIV	91	89	82	81	62	75*	81	51***	61	67	75	74
ILS	98	83*	84	81	63	74*	69	52	40	50	65	68

* *P*<.10; **P<.05; ***P<.01.

Abbreviations: ILS, integrated logistics system; SDP, service delivery point.

^a^ Other management practices comprise design and planning as well as monitoring and control.

^b^ Because of the shift to direct delivery from zonal stores to SDPs at round 1, round-1 storage, inventory management, and transportation scores were absent.

#### District Level

For district-level logistics data management, which focused on management of activities that support collection and dissemination of logistics data, the scores were similar at baseline and round 1 (Table 3). General management scores—which covered whether facility workers received training, whether performance metrics existed and were shared, and whether performance evaluation meetings took place—were also similar, except for HIV commodities, which improved from baseline to round 1 (*P*=.01).

#### Medical Stores Department Zonal and Central Levels

For both HIV and ILS commodities, zonal-level transportation scores improved from the baseline to round 1 across multiple component areas, including use of transport vehicles and adherence to schedules (Table 3). (Use of paired *t* test and a higher significance-level threshold are justified here because almost all the population of the zonal stores was used and some measurement error was assumed.) However, for both HIV and ILS commodities, logistics data management scores deteriorated from the baseline to round 1, as a result of lower performance related to the collection of issues data from districts and SDPs, and sharing of data from the zones with the MSD Central and MOHCDGEC. Storage scores for ILS commodities decreased from baseline to round 1, which was driven by lower performance across multiple component areas. Scores for additional program management practices—including quantification; design and planning; and monitoring and control—showed a greater than 10-point improvement in scores between the baseline and round 1 for 6 of the 10 comparisons.

#### Super Index Management Scores

Combining scores across management domains showed a small improvement from baseline to round 1: for HIV products, scores increased, on average, from 71 to 73; for ILS, from 63 to 67; and for HIV and ILS combined, from 67 to 70. The improvement happened at all tiers of the supply chains and for all commodity groups, except HIV commodities at the zonal stores, which showed a small decrease.

### Supply Chain Outcomes

#### Inventory Availability

**FIGURE 2:**
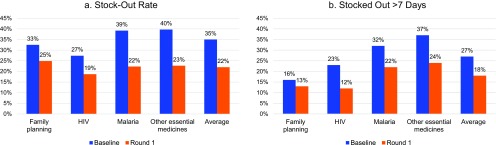
Stock-out Rates and Stock-Out Duration Greater Than 7 Days, Baseline Versus Round 1, by Product Group

Stock-out rates decreased for all 4 product groups by 13 percentage points, on average, from 35% to 22% (Figure 2). Using logistic regression, controlling for delivery groups for ILS facilities, ILS subcommodities, and zone, the odds of stocking out fell by 49% from baseline to round 1 (*P*<.001). Using ordinary least square regression with the same controls, the odds of having stock-outs fell by 13.1 percentage points from baseline to round 1. The duration of stock-outs, measured by the percentage of facilities reporting stock-outs of greater than 7 days, also decreased substantially for all 4 product groups, by about 10 percentage points between baseline and round 1. On average, the percentage of facilities with stock-outs greater than 7 days dropped from 27% to 18% (Figure 2). Using logistic regression, controlling for delivery groups for ILS facilities, ILS subcommodities, and zones, the odds of stocking out for greater than 7 days fell by 44%, from baseline to round 1 (*P*<.001). Using ordinary least square regression with the same controls, the odds of having stock-outs for greater than 7 days fell by 10 percentage points from baseline to round 1. (Logistic regression is the more appropriate model for regression with percentages as dependent variables. However, ordinary least square regression was used to provide more accessible interpretation of the impact of introducing the upgraded system.)

Stock-out rates and duration of stock-outs decreased significantly for all 4 product groups.

#### Inventory Levels

The upgraded system maintained levels of appropriate inventory very similar to what we saw before the upgrade (20% versus 18%). Similarly, levels of high inventory were 28% at round 1 versus 25% at baseline. This suggests that improvement in stock-out performance was not because the facilities were holding higher levels of stock.

#### Forecasting Accuracy

The evaluation revealed a statistically significant decrease between baseline and round 1 in forecast error for family planning commodities, 177% versus 23%. The forecast error for HIV commodities was 33% versus 27%. Forecast error for malaria commodities increased between baseline and round 1, from 12% to 28%. For all commodities combined, we found a statistically significant decrease in forecast error, from 135% to 24% (*P*<.001).

Forecasting accuracy improved overall, especially for family planning commodities.

#### Expiries

Expiry rates at the central and zonal levels did not change noticeably overall (2.39% to 2.45%) (Table 4). SDP expiry rates fell significantly by 0.6 percentage points (*P*<.001), with a lower bound of 0.15 percentage points (data not shown).

**TABLE 4.** Average Central and Zonal Expiry Rates as Percentage of Throughput, Baseline Versus Round 1

Product Group	Expiry Rate (%)	
	Baseline	Round 1
HIV	1.17	0.12
Essential medicines	3.36	3.58
Family planning and MCH	2.45	1.46
Malaria	2.66	3.41
ILS	3.10	3.44
**Average**	**2.39**	**2.45**

Abbreviations: ILS, integrated logistics system; MCH, maternal, newborn, and child health.

### Annual Cost

#### Total Cost

The annual national cost of public health supply chain operations was TSh 62 billion (US$40 million) at baseline; it increased by about 7% to TSh 67 billion (US$43 million) at round 1 (Table 5). Approximately US$1.7 million of the cost increase was the cost of the upgrades; the rest of the increase was probably the result of the higher throughput handled by the system. Annual commodity throughput, adjusted for price changes, increased by 23% from TSh 242 billion (US$154 million) at baseline to TSh 298 billion (US$190 million) at round 1.

**TABLE 5.** Supply Chain Costs by Main Supply Chain Function, Baseline Versus Round 1

Supply Chain Function	TSh in billions (US$ in millions)	
	Baseline	Round 1
Procurement	0.4 (0.3)	0.3 (0.2)
Storage	29.7 (18.9)	31.7 (20.2)
Transport	12.0 (7.6)	11.1 (7.0)
Management	20.2 (12.9)	23.8 (15.1)
**Total**	**62.3 (39.7)**	**66.9 (42.6)**

#### Cost Breakdowns

Of the 4 main supply chain functions, storage had the highest cost, followed by management, transport, and procurement (Table 5). MSD headquarters and zonal stores were the largest contributors to cost, 32% and 27%, respectively, at baseline, and a slightly lower 31% and 25%, respectively, during round 1 (data not shown). District offices were the next-largest tier by cost, accounting for 21% at baseline and 24% during round 1. SDPs followed, making up 17% of the total at baseline and 14% during round 1. The increase in total annual costs between baseline and round 1 came mainly from increases of approximately TSh 3 billion in district-office and development-partner costs.

Average cost per facility in the sample decreased between the baseline and round 1, almost entirely due to a reduction in the cost of managing logistics records (data not shown). Thus, the increase in total annual costs at the national level came primarily from the increase in the number of districts and SDPs served by the supply chain (Table 6).

**TABLE 6.** Comparison in Number of Facilities Served, by Supply Chain Tier, Baseline Versus Round 1

Supply Chain Tier	Baseline	Round 1	% Increase
District	125	164	31%
Dispensary	3,851	4,630	20%
Health center	449	493	10%
District hospital	154	191	24%
Regional hospital	16	21	31%
Referral hospital	4	4	0%

Average cost per facility decreased between the survey rounds, almost entirely due to a reduction in the cost of managing logistics records.

#### Cost-Effectiveness

Before adjusting for performance improvements, cost as a percentage of throughput value at round 1 was lower than at baseline: 22.5% versus 25.7% (Table 7). When adjusting for the observed performance improvement, the gap was even larger: 28.5% at round 1 versus 37.9% at baseline.

**TABLE 7.** Cost-Effectiveness Comparison Measures

Measure	Baseline	Round 1
Supply chain cost (TSh)	62.3 billion	66.9 billion
Value of throughput (TSh)	242 billion	298 billion
% point product availability	68	78
Supply chain cost as % of value of throughput	25.7%	22.5%
Supply chain cost per performance-adjusted throughput value	37.9%	28.5%

#### Sensitivity Analysis

The sensitivity analysis found overlap in the baseline and round 1 95% confidence intervals for total cost and cost per product value (unadjusted) (Table 8). For total value and cost-per-product value (performance-adjusted), no overlap was seen in the confidence intervals.

**TABLE 8.** Results of Sensitivity Analysis Using Monte Carlo Simulation for Cost, Throughput, and Cost-Effectiveness Measures

Cost, Throughput, and Cost-Effectiveness Measures	Baseline		Round 1	
	Mean (SD)	95% CI	Mean (SD)	95% CI
Total cost (Tsh billion)	62.6 (2.2)	58.3, 67.0	69.0 (3.1)	62.9, 75.1
Total throughput value^a^ (TSh billion)	242.4 (5.6)	231.4, 253.3	297.7 (7.3)	283.4, 311.9
Cost as a percentage of product value, unadjusted	25.9% (1.1%)	23.7%, 28.0%	23.2% (1.2%)	20.9%, 25.5%
Cost as a percentage of product value, performance-adjusted^a^	38.2% (1.8%)	34.6%, 41.7%	29.4% (1.6%)	26.3%, 32.4%

Abbreviations: CI, confidence interval; SD, standard deviation.

^a^ Parameter for which there was no overlap in the 95% confidence interval for the baseline and round 1 measures.

### Cost-Benefit Analysis

#### Investment Costs

The total start-up investment cost of the LMU and eLMIS through July 2014 was US$2.4 million (Table 9). The ongoing operational cost totaled US$2.9 million in year 1, which is projected to rise slightly in subsequent years. Early-stage investments of about US$1.2 million benefiting any country were not included in the upfront costs for Tanzania.

**TABLE 9.** Upfront Investment and Ongoing Operating Costs of the eLMIS and LMU (US$)

Category	Actual		Projected			
	Start-up	Year 1	Year 2	Year 3	Year 4	Year 5
**Total eLMIS and LMU**	**2,358,278**	**2,867,981**	**3,041,408**	**2,958,068**	**2,862,776**	**2,922,023**
**Total eLMIS**	**1,768,395**	**698,110**	**772,552**	**633,727**	**481,102**	**481,102**
Development and rollout	1,768,395					
Operations		698,110	772,552	633,727	481,102	481,102
**Total LMU**	**589,883**	**2,169,871**	**2,268,856**	**2,324,341**	**2,381,674**	**2,440,921**
Design	124,693	—	—	—	—	—
Project implementation and technical assistance	208,279	—	30,000	30,000	30,000	30,000
Existing staffing, supervision, training^a^	—	1,206,032	1,237,960	1,270,847	1,304,720	1,339,609
Incremental staffing	—	533,758	549,771	566,264	583,252	600,749
Incremental supervision and training	114,215	264,322	264,027	264,027	264,027	264,027
Vehicles, transport, equipment, etc.	—	91,687	101,760	107,866	114,338	121,198
Office space, equipment, supplies, utilities for LMU	142,696	74,072	85,338	85,338	85,338	85,338

Abbreviations: eLMIS, electronic logistics management information system; LMU, logistics management unit.

^a^ Personnel and supervision/training activities that were included in the organization's budget before the management upgrades were implemented.

#### Operational Savings and Return on Investment

Reduced drug purchase prices; lower expiry rates; and absorption of existing staff, supervision, and training costs generated a savings of US$2.5 million in the first year, after upgrades. Savings are projected to increase to US$3.1 million by year 5 (Table 10). The simple return on investment ratio was negative. However, it is projected to trend positive over time (Table 10).

**TABLE 10.** Estimated Cost Savings and Return on Investment After Implementation of the Upgraded Management System (US$)

	Year 1	Year 2	Year 3	Year 4	Year 5
**Improved pricing for products purchased**					
Annual government drug purchases	47,714,539	61,782,129	70,716,350	79,651,204	88,586,058
Average drop in drug costs from better management practices	668,004	864,950	990,029	1,115,117	1,240,205
**Reduced product waste from expiry**					
Current annual drug throughput subject to expiry	166,707,098	123,202,782	134,928,853	145,538,815	155,590,406
Change in expiry rate after better management practices	625,152	462,010	505,983	545,771	583,464
**Summary cost savings**					
Estimated cost savings due to better management	1,293,155	1,326,960	1,496,012	1,660,887	1,823,669
Absorbed existing staff, supervision, and training	1,206,032	1,237,960	1,270,847	1,304,720	1,339,609
**Total estimated cost savings**	2,499,187	2,564,921	2,766,859	2,965,607	3,163,278
**Total costs**	2,867,981	3,041,408	2,958,068	2,862,776	2,922,023
**Total estimated net savings (savings minus costs)**	(368,794)	(476,487)	(191,209)	102,831	241,255
**Estimated cumulative savings**	(2,727,072)^a^	(3,203,559)	(3,394,768)	(3,291,937)	(3,050,682)
**Simple return on investment**	−52%	−39%	−30%	−23%	−18%

^a^ Includes US$2,358,278 in start-up costs.

Operational savings of US$2.5 million were seen in the first year.

#### Sensitivity Analyses

Projected savings do not assume potential increases in savings rates based on continuous improvement, nor do they include areas of potential savings if data were not available during the study. Sensitivity analyses that used the high range for the expected savings, and assumed a small increase in savings rates based on continuous improvement, produced a positive return on investment by year 5 (data not shown).

## DISCUSSION

This study examined the effects of introducing supply chain upgrades—the LMU and eLMIS—to the public health supply chain of Tanzania. The upgrades were associated with a positive impact on key supply chain outcomes, especially stock-out rates and stock-out duration. Encouragingly, the decrease in stock-outs did not appear to happen because of increased overstocking. The upgrades were also associated with a large relative decrease in expiry rates.

Results from the analysis of data-use and management practices gave additional evidence of a causal link. Improvements in data use, accessibility, visibility, and transparency, as well as improvements in planning, control, and monitoring and support for quantification, may have resulted directly from the LMU's efforts to consolidate oversight and improve management efficiency. Management practices beyond the organizational boundaries of the LMU and supply chain infrastructure changed little, perhaps because the lead time for such downstream improvements is longer than the 1-year duration of this study.

The explanation for the positive results was multilayered, as expected by the designers of the upgrades. One year after their implementation, the upgrades appear to have affected supply chain performance primarily through better data use and through improvements in some, but not all, management practices. Through its increase in zonal personnel and an expansion of its mandate to include commodity groups beyond HIV, the LMU also may have had a direct influence on supply chain outcomes at the district and facility levels.

The upgraded system was more costly but also more efficient, particularly when adjusting for the performance improvements. The upgrades also generated substantial savings that defrayed some, but not all, of the investment costs. Placed next to the improvements in supply chain performance, these savings were a substantial “bonus.”

Substantial savings from the upgrades defrayed some of the investment costs.

Notably, observed improvements happened during a challenging period: MSD debt levels increased at this time, which further hampered the organization's operations. Major shifts in procurement modalities and global availability affected stock levels of some antiretrovirals and antimalarials.

### Limitations

The study results have some important methodological limitations. First, the national rollout of the LMU and eLMIS precluded randomization. Some changes to the supply chain distribution structure occurred shortly before or during implementation of the management upgrades. We tried to mitigate this limitation by considering factors other than the introduction of the eLMIS and LMU that might have influenced supply chain performance and cost. Although it is impossible to completely disentangle the impact of all of these factors, our comprehensive measurement of performance across different functions of the supply chain provided us with sufficient insight to trace the mechanisms that connect these factors to improving supply chain outcomes.

Second, although we based composite reporting, data-use, and management practice indicators on previously well-established methods, it was a subjective exercise that introduced possibilities for error or bias. Furthermore, to the extent that our estimate of national supply chain cost relied on survey data, true national costs may differ from our estimates. Despite training, simplification of data collection forms, and quality control, the survey methodology introduced possibilities for errors that may affect the robustness of the cost results. The sensitivity analyses, in part, addressed these limitations.

Finally, the evaluation examined young and still-maturing interventions. Thus, it is entirely reasonable to expect that the full impact of these investments will only be seen in 2, 3, or even 5 years after their initial rollout.

## CONCLUSIONS

The results confirmed the designers' expectations that management upgrades would create multiple and complex pathways to impact. One year after implementation of upgrades to key supply chain systems, the LMU and eLMIS appeared to have worked primarily through better data use and through improvements in some, but not all, management practices. Furthermore, the upgrades—while not inexpensive—contributed to greater system efficiency and modest savings.

## Supplementary Material

Supplement 1

## Notes

**Peer Reviewed**

**First Published Online:** 2017 September 5
