# Supplementary material for: Upgrading Supply Chain Management Systems to Improve Availability of Medicines in Tanzania: Evaluation of Performance and Cost Effects
Source: Glob Health Sci Pract. 2017 Sep 27;5(3):399–411. doi: 10.9745/GHSP-D-16-00395 (PMC5620337; doi:10.9745/GHSP-D-16-00395)
Supplement: Supplement 1 [file GHSP-D-16-00395_index.html]

Supplement to Upgrading Supply Chain Management Systems to Improve Availability of Medicines in Tanzania: Evaluation of Performance and Cost Effects | Global Health: Science and Practice

## Supplemental material

- Text s01, PDF - Text s01, PDF
- Text s02, PDF - Text s02, PDF
- Text s03, PDF - Text s03, PDF
