# Supplementary material for: Upgrading Supply Chain Management Systems to Improve Availability of Medicines in Tanzania: Evaluation of Performance and Cost Effects
Source: Glob Health Sci Pract. 2017 Sep 27;5(3):399–411. doi: 10.9745/GHSP-D-16-00395 (PMC5620337; doi:10.9745/GHSP-D-16-00395)
Supplement: Supplement 1 [file 16-00395-Rosen-Supplement1.pdf]

Mwencha M, Rosen JE, Spisak C, Watson N, Kisoka N, Mberesero H. Upgrading supply chain management systems to improve availability of medicines in Tanzania: evaluation of performance and cost effects. *Glob Health Sci Pract*. 2017;5(3). https://doi.org/10.9745/GHSP-D-16-00395

SUPPLEMENT 1. Tracer Commodities Included in the Study

Product Group (Subgroup)	Tracer Commodity
HIV	Duovir N (AZT300/3TC150/NVP200)<br>TLE (TDF300/3TC300/EFV600)<br>Atripla (TDF300/FTC200/EFV600)<br>Combivir (AZT300/3TC150)<br>Efavirenz 600 mg<br>Nevirapine 200 mg<br>Nevirapine syrup<br>Alere Determine HIV-1/2 test kit<br>Uni-Gold test kit<br>Dried blood spot kit
ILS (family planning and MNCH)	Condoms<br>Depo-Provera<br>Implants<br>Intrauterine devices<br>Progestin-only (Microval/Microlut) pill<br>Combined oral (Microgynon) pill<br>Oxytocin injection<br>Misoprostol tablets<br>Magnesium sulphate injection<br>Oral rehydration solution sachet<br>Ferrous folic acid (FeFol) tablet<br>Albendazole tablet (200 mg)
ILS (Malaria)	ALu 1x6 (strip of 6)<br>ALu 2x6 (strip of 12)<br>AL 3x6 (strip of 18)<br>AL 4x6 (strip of 24)<br>Sulphadoxine/pyrimethamine tablet<br>Artesunate injection<br>Quinine tablet<br>Quinine injection<br>Malaria rapid diagnostic test
ILS (Other essential)	Diazepam injection<br>Amoxicillin (250 mg) capsule<br>Amoxicillin (suspension)<br>Paracetamol (500 mg) tablet<br>Cotrimoxazole (480 mg) tablet<br>Cotrimoxazole (suspension)<br>Dextrose 5% (500 ml)

Abbreviations: ILS, integrated logistics system; MNCH, maternal, neonatal, and child health.
