# Supplementary material for: Upgrading Supply Chain Management Systems to Improve Availability of Medicines in Tanzania: Evaluation of Performance and Cost Effects
Source: Glob Health Sci Pract. 2017 Sep 27;5(3):399–411. doi: 10.9745/GHSP-D-16-00395 (PMC5620337; doi:10.9745/GHSP-D-16-00395)
Supplement: Supplement 1 [file 16-00395-Rosen-Supplement2.pdf]

Mwencha M, Rosen JE, Spisak C, Watson N, Kisoka N, Mberesero H. Upgrading supply chain management systems to improve availability of medicines in Tanzania: evaluation of performance and cost effects. *Glob Health Sci Pract*. 2017;5(3).
https://doi.org/10.9745/GHSP-D-16-00395

SUPPLEMENT 2. Calculation of Expiry Rate

Expiries at zonal stores and the central Medical Stores Department were analyzed using data from commodities expiring with manufacturer-provided expiry dates from January 2013 to May 2015. These data covered both the value and quantity of the expiring commodities. Inventory transaction data from zonal stores during the same period complemented the expiry data and allowed calculation of throughput to normalize expiries. We used both data sets to compare the expiries as a percentage of annual throughput during a baseline period (for expiry analysis considered between January and December 2013) and round 1 (from April 2014 to March 2015). Although the database identified commodities as expiring at the manufacturer-provided expiry date, the reality is that these expiries resulted from actions taken before that final date. We refer to the time before the expiry date where actions would most determine final expiries as the *period of responsibility* and attribute expiries to the baseline or round 1, based on the length of the overlap of the period of responsibility with those time periods, respectively. To generate an average, we allow this period of responsibility to vary from 3 months to 9 months. Specifically, to calculate expiries, we define the *daily expiration rate* as the value of commodities expiring divided by the period of responsibility in days. We then calculated expiries in the baseline and round 1 period as the product of the daily rate and the number of days in the overlap of the period of responsibility and the baseline and round 1 (see Figure, next page).

Expiries at the service delivery point level draw on 2 data sets. The first were the data from the End-Use Verification (EUV) visits at the baseline (August 2013) and at round 1 (April 2015). Expiry rates were defined as product expiries as a percentage of consumption in the previous 3 months for baseline and round 1. Expiry rates higher than 200% were considered outliers. The EUV surveys are applied to a different sample of facilities each quarter, chosen at random as representative of the entire population of facilities. One limitation of this approach is that the timing of these expiries is unknown. Ideally, the expiries should have occurred during the prior 3 months, but that may not have been the case.

The second data set used to examine expiries was the time series data from 12 quarterly EUV visits from May 2012 to April 2015 (except February 2012), which were used to analyze inventory availability. Again, these EUV visits only focused on integrated logistics system (ILS) commodities. The analysis allowed for examining the effect of introducing the electronic logistic management information system (eLMIS) (considered to impact the last 3 quarters, starting October 2014) and the training of zonal Logistics Management Unit (LMU) staff (considered to impact the last 6 quarters, starting February 2014) on expiry rates—again defined as product expiries as a percentage of consumption in the previous 3 months. The impact of these events could be analyzed, controlling for the impact of trend or seasonality over the 3 years and the sub-commodity groups—malaria; family planning and maternal, newborn, and child health; and essential medicines. Using generalized linear model (Poisson), introduction of the eLMIS was found to reduce the average expiry rate by 0.6% of the expiry rate ($P = 1.54 \times 10^{-5}$), but LMU zonal staff training had no significant effect ($P = 0.854$). Using ordinary least square regression, the introduction of the eLMIS reduced the average expiry rate by 0.6 percentage points. Since the timing of the expiries in this dataset is unknown, if we assume that expiries identified in

Mwencha M, Rosen JE, Spisak C, Watson N, Kisoka N, Mberesero H. Upgrading supply chain management systems to improve availability of medicines in Tanzania: evaluation of performance and cost effects. *Glob Health Sci Pract*. 2017;5(3). https://doi.org/10.9745/GHSP-D-16-00395

the dataset occur or renew quarterly, this reduction in expiries by 0.6% of consumption is an upper bound on the actual impact of the eLMIS introduction. At the other extreme, if we assume that the expiries identified actually occur or renew annually, the reduction in expiries by the eLMIS introduction has a lower bound of 0.15% of consumption.

FIGURE. Estimating Expiries at Zonal and MSD Central Stores

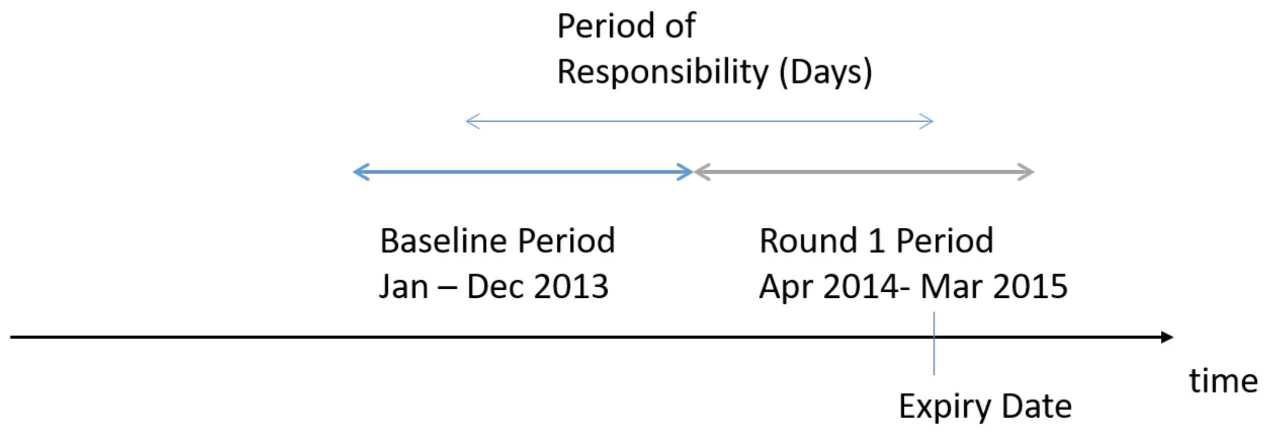

- Daily Expiration Rate = Expiries (at expiry date)/Period of responsibility
- Expiries in Pre-ELMIS Period = Expiration rate x Overlap in baseline period and period of responsibility (days)
- Expiries in ELMIS Period = Expiration rate x Overlap in Round 1 period and period of responsibility (days)
- Expiries in each period can be expressed as a percentage of throughput.
- Period of responsibility can be varied. We varied from 3 months to 9 months to get an average.

Abbreviations: eLMIS, electronic logistics management information system; MSD, Medical Stores Department.
