# Supplementary material for: Upgrading Supply Chain Management Systems to Improve Availability of Medicines in Tanzania: Evaluation of Performance and Cost Effects
Source: Glob Health Sci Pract. 2017 Sep 27;5(3):399–411. doi: 10.9745/GHSP-D-16-00395 (PMC5620337; doi:10.9745/GHSP-D-16-00395)
Supplement: Supplement 1 [file 16-00395-Rosen-Supplement3.pdf]

Mwencha M, Rosen JE, Spisak C, Watson N, Kisoka N, Mberesero H. Upgrading supply chain management systems to improve availability of medicines in Tanzania: evaluation of performance and cost effects. *Glob Health Sci Pract*. 2017;5(3).
https://doi.org/10.9745/GHSP-D-16-00395

SUPPLEMENT 3. Methodology for Sensitivity Analysis

We identified the throughput, cost, and performance parameters holding a substantial degree of uncertainty (see Table, next page). For each parameter, we assumed an underlying normal distribution of possible values. We defined our base case assumption as the mean of this distribution and assigned a standard deviation, minimum, and maximum based on either (1) a reasonable notion of the range of possible values that the parameter might take, or (2) on the 95% confidence interval for the sample parameter distribution. Next, for the baseline and round 1 values, we allowed each of these parameters, simultaneously, to vary randomly; each time calculating a total cost, total throughput, performance, and associated cost and cost-effectiveness measures. We repeated this procedure 10,000 times and derived a mean, standard deviation, and 95% confidence intervals for cost and cost-effectiveness measures.

Mwencha M, Rosen JE, Spisak C, Watson N, Kisoka N, Mberesero H. Upgrading supply chain management systems to improve availability of medicines in Tanzania: evaluation of performance and cost effects. *Glob Health Sci Pract*. 2017;5(3). https://doi.org/10.9745/GHSP-D-16-00395

TABLE. Throughput and Cost Parameters Used in the Sensitivity Analysis (US$)

Parameter	Mean (base)	SD	Minimum	Maximum
Storage cost	6.00	1.00	5.00	10.00
**SDP storage space estimates, baseline (m^2)**				
Dispensary	12.00	31.24	9.60	14.40
Health center	20.00	35.53	12.40	27.60
District hospital	56.00	62.15	14.56	97.44
**SDP storage space estimates, round 1 (m^2)**				
Dispensary	12.00	13.71	10.56	13.44
Health center	20.00	16.48	14.20	25.80
District hospital	132.00	21.07	108.24	155.76
**Supply chain performance**				
Stock-out rate, baseline				
EPI baseline performance	23%	45%	18%	27%
HIV baseline performance	27%	45%	25%	30%
Other ILS baseline performance	40%	49%	38%	42%
Malaria baseline performance	39%	49%	37%	41%
Family planning baseline performance	32%	47%	30%	35%
Stock-out rate, round 1				
HIV round 1 performance	19%	39%	17%	21%
Other ILS round 1 performance	23%	42%	21%	25%
Malaria round 1 performance	22%	42%	20%	24%
Family planning round 1 performance	25%	43%	23%	27%
**SDP, district, and regional costs**				
All surveyed SPD, district, and regional costs, baseline	Sample mean	Sample SD	95% CI around mean	95% CI around mean
All surveyed SDP, district, and regional costs, round 1	Sample mean	Sample SD	95% CI around mean	95% CI around mean
	**Mean**	**SD**	**Min value as % of base value**	**Max value as % of base value**
Throughput value	0%	4%	-10%	10%

Abbreviations: CI, confidence interval; EPI, Expanded Programme on Immunization; ILS, integrated logistics system; SD, standard deviation; SDP, service delivery point.
